# Supplementary material for: The Sensory-Directed Elucidation of the Key Tastants and Odorants in Sourdough Bread Crumb
Source: Foods. 2022 Aug 4;11(15):2325. doi: 10.3390/foods11152325 (PMC9368349; doi:10.3390/foods11152325)
Supplement: Supplementary file 1 [file foods-11-02325-s001.zip › foods-1812906-supplementary.pdf]

Supporting Information

The Sensory-Directed Elucidation of the Key Tastants and Odorants in Sourdough Bread Crumb

Laura Sophie Amann, Oliver Frank*, Corinna Dawid, and Thomas Frank Hofmann

Food Chemistry and Molecular Sensory Science, TUM School of Life Sciences, Technical University of Munich, Lise-Meitner-Straße 34, 85354 Freising, Germany

*** Author to whom correspondence should be addressed**

PHONE +49-8161-712910

FAX +49-8161-712949

E-MAIL oliver.frank@tum.de

Methodology: LC-MS/MS Quantitation of Basic Taste Compounds

1. Free Amino Acids

The quantitative analysis of free amino acids was conducted using SIDA by means of UHPLC-MS/MS as reported earlier [29,30] with the following modifications. Aliquots (1 µL) of the sample extracts were injected into a Nexera X2 UHPLC system (Shimadzu, Duisburg, Germany) connected to a 5500 QTRAP MS/MS system (Sciex) operating in positive electrospray ionization (ESI^+) mode. The source temperature was set to 500 °C. The chromatographic separation was carried out at 40 °C using an Acquity BEH amide column (2.1 mm × 100 mm, 1.7 µm; Waters, Eschborn, Germany) with a corresponding guard column (2.1 mm × 5 mm; Waters) at a flow rate of 0.4 mL/min. The eluent system consisted of ammonium acetate (5 mM) in water (A) and ammonium acetate (5 mM) in acetonitrile/water (95/5, *v*/*v*; B), both adjusted to pH 3.0 with acetic acid. The gradient elution started with 10% A which was increased to 15% within 5 min and to 45% within the next 3 min. Then, eluent A was raised to 100% within 1 min and maintained at this level for 2 min. The regeneration of the starting conditions (10% A) was performed within 1 min followed by isocratic elution for further 2 min.

2. Glutamyl Dipeptides

The quantification of α- and γ-glutamyl dipeptides was carried out using an UltiMate 3000 UHPLC system (Dionex) connected to an API 4000 QTRAP MS/MS system (Sciex) operating in ESI^+ mode as reported earlier [23,31] with some modifications. The ion source parameters were the following: curtain gas (30 psi), source temperature (425 °C), nebulizer gas (55 psi), heating gas (65 psi), collision activated dissociation (-2 V), and entrance potential (10 V). The chromatographic separation was performed at 40 °C with a flow rate of 0.2 mL/min by injecting aliquots (5 µL) of the sample extracts into the UHPLC system equipped with a Luna PFP column (2 mm × 150 mm, 3 µm; Phenomenex) and a corresponding guard column. The gradient elution was conducted with formic acid (1%) in water (A) and formic acid (1%) in acetonitrile (B), starting with 100% A for 5 min. Eluent A was decreased to 30% within 11 min, to 0% within the next 1 min, and maintained at 0% for further 2 min. The starting conditions (100% A) were re-established within 1 min followed by isocratic elution for further 5 min.

3. Nucleotides and Nucleosides

Following a literature protocol [23] with several modifications, the nucleotides and nucleosides were quantified using SIDA by means of an UltiMate 3000 UHPLC system (Dionex) connected to an API 4000 QTRAP MS/MS system (Sciex). Running in negative electrospray ionization (ESI^-) mode, the ion source parameters were as follows: curtain gas (35 psi), source temperature (450 °C), nebulizer gas (55 psi), heating gas (65 psi), collision activated dissociation (-2 V), and entrance potential (-10 V). Aliquots (5 µL) of the sample extracts were injected into the UHPLC system equipped with a XBridge amide column (2.1 mm × 150 mm, 3.5 µm; Waters) with a corresponding guard column. Chromatography was performed at a flow rate of 0.3 mL/min at 50 °C with an eluent system consisting of ammonium acetate (5 mM) in acetonitrile/water (50/50, *v*/*v*; A) and ammonium acetate (5 mM) in acetonitrile/water (95/5, *v*/*v*; B), both adjusted to pH 9.0 with ammonium hydroxide. The initial conditions (0.1% A) of the gradient elution were maintained for 0.5 min. Then, eluent A was increased to 40% within 2.6 min, to 70% within the next 10.9 min, to 100% within 2 min, and kept at this level for further 2 min. In the end, eluent A was reduced to 0.1% within 1 min and held at this level for 4 min.

4. Organic Acids

The quantitative analysis of low molecular weight organic acids was performed using an UltiMate 3000 UHPLC system (Dionex) connected to an API 4000 QTRAP MS/MS system (Sciex) operating in ESI^- according to Mittermeier et al. [22] with the following modifications. The following ion source

parameters were used: curtain gas (40 psi), source temperature (700 °C), nebulizer gas (55 psi), heating gas (65 psi), collision activated dissociation (-2 V), and entrance potential (-10 V). The chromatographic separation was performed at 40 °C with a flow rate of 0.5 mL/min by injecting aliquots (5 µL) of the sample extracts into the UHPLC system equipped with a ZIC-pHILIC column (4.6 mm × 150 mm, 5 µm; Merck) and a corresponding guard column. Ammonium acetate (5 mM) in water (pH 9.5) was used as eluent A and ammonium acetate (5 mM) in acetonitrile/water (95/5, *v*/*v*, pH 9.5) as eluent B. After 4 min of isocratic elution (20% A), eluent A was increased to 100% within 8 min and this level was retained for the next 6 min. Then, eluent A was decreased to 20% within 1 min and remained at this level for further 6 min.

5. Quaternary Ammonium Compounds and Opines

The quaternary ammonium compounds (QAC) and opines were quantified using SIDA by means of UHPLC-MS/MS according to a procedure reported earlier [23,24] with some modifications. Aliquots (1 µL) of the sample extracts were injected into a Nexera X2 UHPLC system (Shimadzu) connected to a QTRAP 6500 MS/MS system (Sciex) running in ESI^+ mode. The following instrument settings were used: curtain gas (50 psi), source temperature (550 °C), nebulizer gas (55 psi), heating gas (65 psi), collision activated dissociation (-2 V), and entrance potential (10 V). The QACs and opines were separated at 50 °C using an Acquity BEH amide column (2.1 mm × 100 mm, 1.7 µm; Waters) with a corresponding guard column at a flow rate of 0.5 mL/min. Water containing ammonium acetate (15 mM) and formic acid (1.25%) was used as eluent A and acetonitrile/water (95/5, *v*/*v*) with ammonium acetate (15 mM) and formic acid (1.25%) as eluent B. Starting with 5% A for 1.5 min, eluent A was increased to 27.5% within 2 min and to 55% within the next 1.5 min. Afterwards, it was raised to 70% within 0.75 min and maintained at this level for further 0.75 min. Finally, the starting conditions (5% A) were re-established within 1 min and retained for 2.5 min.

(1) Glycerol + ATP $\xrightarrow{\text{Glycerokinase}}$ L-Glycerol-3-phosphate + ADP

(2) ADP + phosphoenolpyruvate $\xrightarrow{\text{Pyruvate kinase}}$ ATP + Pyruvate

(3) Pyruvate + NADH + H^+ $\xrightarrow{\text{L-Lactate dehydrogenase}}$ L-Lactate + NAD^+

Figure S1. Enzymatic reactions for the determination of glycerol including adenosine-5'-triphosphate (ATP) and adenosine-5'-diphosphate (ADP).

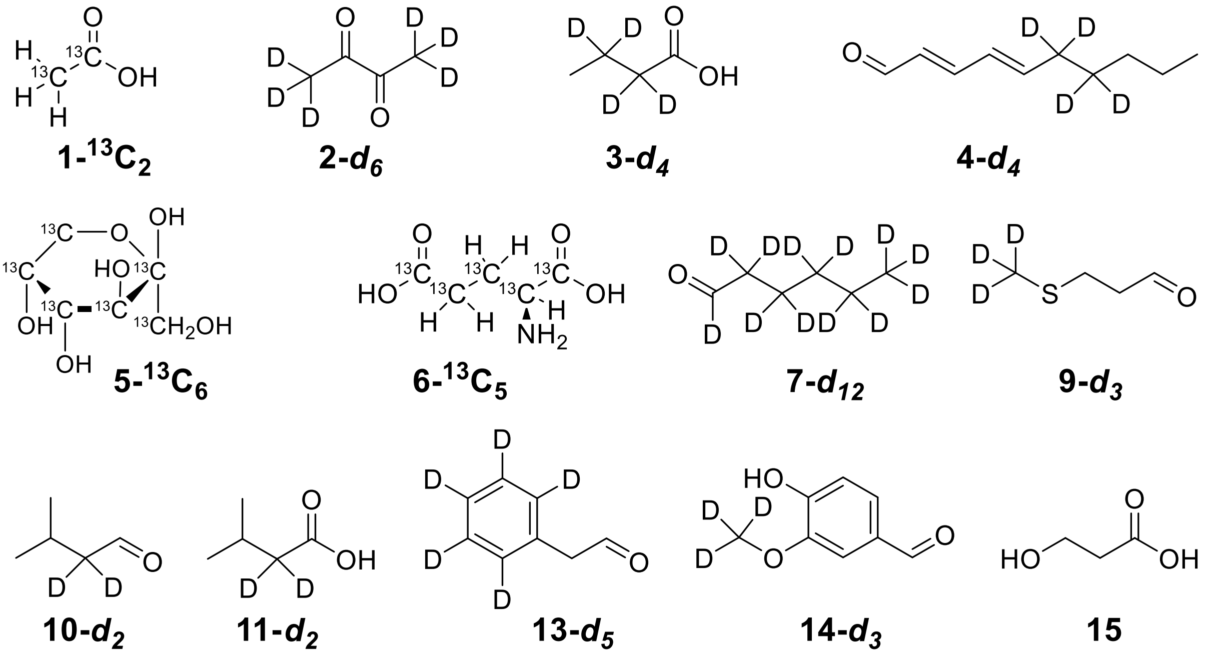

Figure S2. Chemical structures of internal standards used for quantification of key taste and aroma compounds in bread crumb: acetic acid-$^{13}C_2$ (**1**-$^{13}C_2$), 2,3-butanedione-d_6 (**2**-d_6), butyric acid-d_4 (**3**-d_4), (*E*,*E*)-2,4-decadienal-d_4 (**4**-d_4), D-fructose-$^{13}C_6$ (**5**-$^{13}C_6$), L-glutamic acid-$^{13}C_5$ (**6**-$^{13}C_5$), hexanal-d_{12} (**7**-d_{12}), methional-d_3 (**9**-d_3), 3-methylbutanal-d_2 (**10**-d_2), 3-methylbutyric acid-d_2 (**11**-d_2), phenylacetaldehyde-d_5 (**13**-d_5), vanillin-d_3 (**14**-d_3), and 3-hydroxypropionic acid (**15**).

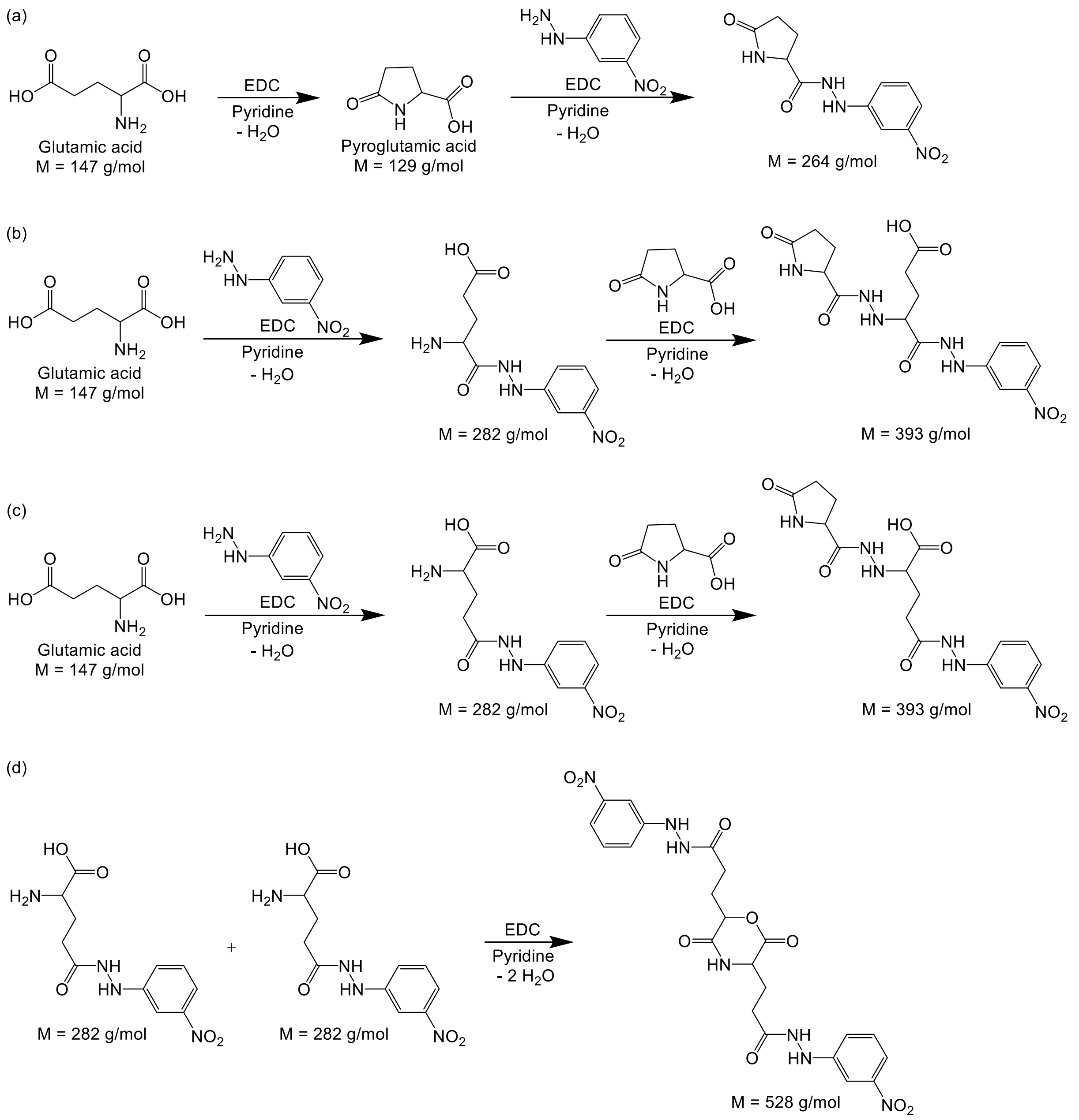

Figure S3. Overview of reactions taking place during the derivatization of glutamic acid with 3-nitrophenylhydrazine (3-NPH) using *N*-(3-(dimethylamino)propyl)-*N′*-ethylcarbodiimide (EDC) as a coupling reagent and pyridine as a catalyst (40 °C, 30 min). The predominant reaction is reaction (**a**).

Table S1. Concentration of aroma compounds in total aroma recombinant of a sourdough rye bread crumb with odor activity values in water (OAV_{water}) and starch (OAV_{starch}) adapted with permission from [2]. Copyright 2001 American Chemical Society.

Aroma Compound	Odor Quality	Concentration [µg/kg]	OAV_{water}	OAV_{starch}
Acetic acid	Sour, pungent	1700000	77.3	54.6
3-Methylbutanol	Malty	2800	2.8	27.5
Butyric acid	Sweaty	2200	2.2	22.0
Vanillin	Vanilla-like	1200	48.0	261
3-Methylbutyric acid	Sweaty	1100	1.5	200
Phenylacetic acid	Honey-like	741	0.07	2.8
2-Phenylethanol	Flowery	716	0.7	5.7
Hexanal	Green, grassy	380	36.2	12.7
2,3-Butanedione	Buttery	334	22.3	51.4
Phenylacetaldehyde	Honey-like	244	61.0	8.7
3-Methylbutanal	Malty	150	375	4.7
Methional	Cooked potato-like	75	41.7	278
(*E*,*E*)-2,4-Decadienal	Fatty, waxy	62	310	23.0
(*E*)-2-Nonenal	Green, fatty	49	61.3	92.5
2,3-Pentanedione	Buttery	43	1.4	2.7
Sotolon	Spicy	6.3	21.0	3.0
(*Z*)-4-Heptenal	Fatty	2.7	13.5	0.9

Table S2. Taste profile analysis (TPA) of the extracts and recombinants of the rye bread crumb, mixed-type bread crumb, and wheat bread crumb.

Name[a]	Intensity of Individual Taste Qualities[b]							No. of Panelists
	Salty	Sour	Sweet	Bitter	Astringent	Umami	Kokumi	
RBC	2.43±0.76	2.46±0.77	0.65±0.37	0.91±0.25	0.34±0.23	0.80±0.25	0.33±0.18	16
RBCE	2.95±0.67	2.07±0.83	0.63±0.33	1.02±1.06	0.32±0.39	0.83±0.64	0.36±0.31	15
RBCE$^+$	2.96±0.42	3.17±0.49	0.64±0.40	1.04±0.19	0.33±0.13	0.82±0.20	0.35±0.20	16
tRec$^+_{rye}$(matrix)	2.54±0.69	2.58±0.67	0.60±0.13	0.87±0.17	0.36±0.22	0.75±0.19	0.30±0.16	16
tRec$_{rye}$	3.00±0.69	2.26±0.70	0.69±0.30	0.90±0.48	0.36±0.24	0.85±0.31	0.41±0.25	14
tRec$^+_{rye}$	3.03±0.44	3.22±0.63	0.61±0.28	0.93±0.30	0.33±0.12	0.79±0.21	0.39±0.16	16
pRec$^+_{rye}$(matrix)	2.40±0.32	2.63±0.33	0.58±0.27	0.89±0.29	0.37±0.21	0.72±0.17	0.29±0.15	16
pRec$_{rye}$	2.93±0.74	2.24±0.87	0.68±0.31	0.88±0.25	0.29±0.13	0.77±0.41	0.40±0.15	15
pRec$^+_{rye}$	2.92±0.37	3.26±0.73	0.70±0.19	0.88±0.29	0.24±0.14	0.73±0.40	0.40±0.16	15
MBC	2.54±0.43	2.14±0.65	0.43±0.28	0.57±0.29	0.22±0.17	0.64±0.33	0.23±0.18	17
MBCE	3.39±0.36	1.58±0.35	0.45±0.21	0.65±0.24	0.26±0.13	0.57±0.21	0.27±0.12	16
MBCE$^+$	3.41±0.42	2.54±0.50	0.44±0.22	0.64±0.24	0.27±0.15	0.56±0.22	0.27±0.12	16
tRec$^+_{mixed}$(matrix)	2.50±0.42	2.07±0.50	0.38±0.14	0.53±0.15	0.23±0.13	0.59±0.26	0.19±0.10	16
tRec$^+_{mixed}$	3.48±0.45	2.58±0.58	0.40±0.22	0.61±0.22	0.32±0.22	0.55±0.20	0.25±0.10	16
pRec$^+_{mixed}$(matrix)	2.46±0.39	2.03±0.45	0.40±0.16	0.49±0.14	0.25±0.15	0.55±0.27	0.20±0.11	16
pRec$^+_{mixed}$	3.46±0.37	2.49±0.55	0.39±0.15	0.57±0.27	0.30±0.12	0.55±0.24	0.25±0.10	16
WBC	2.56±0.50	1.47±0.67	0.38±0.30	0.51±0.30	0.20±0.16	0.49±0.34	0.20±0.16	17
WBCE	3.28±0.37	1.31±0.35	0.41±0.20	0.52±0.22	0.23±0.08	0.48±0.22	0.25±0.13	16
WBCE$^+$	3.36±0.26	2.19±0.47	0.40±0.19	0.54±0.23	0.24±0.12	0.46±0.25	0.23±0.11	16
tRec$^+_{wheat}$(matrix)	2.56±0.58	1.52±0.42	0.40±0.20	0.47±0.21	0.21±0.13	0.46±0.23	0.18±0.07	16
tRec$^+_{wheat}$	3.43±0.29	2.42±0.37	0.34±0.11	0.49±0.23	0.27±0.14	0.52±0.18	0.21±0.07	16
pRec$^+_{wheat}$(matrix)	2.49±0.70	1.50±0.40	0.40±0.19	0.53±0.22	0.25±0.16	0.48±0.17	0.17±0.07	16
pRec$^+_{wheat}$	3.49±0.37	2.37±0.48	0.33±0.10	0.45±0.17	0.28±0.10	0.52±0.20	0.24±0.10	16

[a] Taste profile analysis of the rye bread crumb (RBC), the aqueous solution of the lyophilized rye bread crumb extract without (RBCE) and with spiking of acetic and lactic acid (RBCE$^+$), the total basic recombinant of rye bread crumb without spiking in water (tRec$_{rye}$) and with spiking of acetic and lactic acid in water (tRec$^+_{rye}$) and in matrix (tRec$^+_{rye}$(matrix)), the partial basic recombinant of rye bread crumb without spiking in water (pRec$_{rye}$) and with spiking of acetic and lactic acid in water (pRec$^+_{rye}$) and in matrix (pRec$^+_{rye}$(matrix)), the mixed-type bread crumb (MBC), the aqueous solution of the lyophilized mixed-type bread crumb extract without (MBCE) and with spiking of acetic and lactic acid (MBCE$^+$), the total basic recombinant of mixed-type bread crumb with spiking of acetic and lactic acid in water (tRec$^+_{mixed}$) and in matrix (tRec$^+_{mixed}$(matrix)), the partial basic recombinant of mixed-type bread crumb with spiking of acetic and lactic acid in water (pRec$^+_{mixed}$) and in matrix (pRec$^+_{mixed}$(matrix)), the wheat bread crumb (WBC), the aqueous solution of the lyophilized wheat bread crumb extract without (WBCE) and with spiking of acetic and lactic acid (WBCE$^+$), the total basic recombinant of wheat bread crumb with spiking of acetic and lactic acid in water (tRec$^+_{wheat}$) and in matrix (tRec$^+_{wheat}$(matrix)), and the partial basic recombinant of wheat bread crumb with spiking of acetic and lactic acid in water (pRec$^+_{wheat}$) and in matrix (pRec$^+_{wheat}$(matrix)), [b] the perceived intensities of the individual taste qualities were evaluated on a linear scale from 0 (not perceivable) to 5 (very intensely perceivable) and are given as the mean of the rated intensities of all panelists±standard deviation.

Table S3. Concentration of basic taste compounds in sourdough rye bread crumb with 0.6 > DoT ≥ 0.1.

Taste Compound	Concentration (±SD) [mmol/kg]	DoT Factor[a]
**Group II: Bitter-tasting compounds**		
Ethanol	57.8 (±8.7)	0.2
**Group III: Sweet-tasting compounds**		
Betaine	10.7 (±0.1)	0.5
Glycerol	25.5 (±0.2)	0.3
Glucose	14.5 (±0.9)	0.3
Sucrose	3.03 (±0.07)	0.3
Maltose	26.8 (±1.1)	0.3
Maltotriose	4.45 (±0.48)	0.3
Maltotetraose	1.33 (±0.09)	0.1
L-Alanine	0.984 (±0.009)	0.1

[a] DoT factor = dose-over-threshold factors.

Table S4. Omission and combination tests applied to the aroma recombinant of rye bread crumb by means of triangle tests.

Omitted Odorant[a]	Combined Odorants[a]	Aroma Quality	Significance[b,c]	*p* Value[c]	Significance[b,d]	*p* Value[d]
**21**		Flowery	NS	0.791	NS	0.382
**23**		Spicy	NS	0.524	NS	0.310
**24**		Fatty	NS	0.895	NS	0.310
	**2** with **22**	Buttery	NS	0.678	NS	0.448
	**3** with **11**	Sweaty	NS	0.149	**	0.004
	**4** with **12**	Fatty	**	0.009	**	0.002
	**10** with **19**	Malty	NS	0.382	NS	0.596
	**13** with **20**	Honey-like	NS	0.149	NS	0.524
**21**, **23**, and **24**	**2** with **22**, **10** with **19**, and **13** with **20**		NS	0.075	NS	0.172

[a] Names of the numbered analytes: 2,3-butanedione (**2**), butyric acid (**3**), (*E*,*E*)-2,4-decadienal (**4**), 3-methylbutanal (**10**), 3-methylbutyric acid (**11**), (*E*)-2-nonenal (**12**), phenylacetaldehyde (**13**), 3-methylbutanol (**19**), phenylacetic acid (**20**), 2-phenylethanol (**21**), 2,3-pentanedione (**22**), sotolon (**23**), and (*Z*)-4-heptenal (**24**), [b] NS = not significant ($p > 0.05$), ** = highly significant ($0.01 \geq p > 0.001$), [c] the omission test was carried out in citrate buffer solution, [d] the omission test was carried out in starch.

Table S5. MRM transitions of analyzed 3-NPH derivatives in negative electrospray ionization mode.

No.	Name[a]	Q1 [Da]	Q3 [Da]	DP [V]	EP [V]	CE [V]	CXP [V]
1	Acetic acid qn	193.9	151.7	-155	-10	-20	-37
1	Acetic acid ql	193.9	137.0	-155	-10	-26	-17
1-$^{13}C_2$	(IS) Acetic acid-$^{13}C_2$ qn	196.0	152.0	-125	-10	-18	-17
1-$^{13}C_2$	(IS) Acetic acid-$^{13}C_2$ ql	196.0	137.0	-100	-10	-28	-9
2	2,3-Butanedione 2-fach qn	355.0	136.7	-150	-10	-26	-15

2	2,3-Butanedione 2-fach ql	355.0	45.9	-150	-10	-96	-21
2-*d*$_6$	(IS) 2,3-Butanedione-*d*$_6$ 2-fach qn	361.0	137.8	-150	-10	-26	-15
2-*d*$_6$	(IS) 2,3-Butanedione-*d*$_6$ 2-fach ql	361.0	46.0	-150	-10	-96	-21
3	Butyric acid qn	222.0	151.9	-105	-10	-20	-23
3	Butyric acid ql	222.0	136.9	-105	-10	-24	-37
3-*d*$_4$	(IS) Butyric acid-*d*$_4$ qn	226.0	152.9	-75	-10	-22	-15
3-*d*$_4$	(IS) Butyric acid-*d*$_4$ ql	226.0	136.9	-75	-10	-26	-15
4	(*E*,*E*)-2,4-Decadienal qn	286.1	136.9	-135	-10	-28	-15
4	(*E*,*E*)-2,4-Decadienal ql	286.1	46.0	-135	-10	-74	-21
4-*d*$_4$	(IS) (*E*,*E*)-2,4-Decadienal-*d*$_4$ qn	290.0	136.9	-95	-10	-28	-15
4-*d*$_4$	(IS) (*E*,*E*)-2,4-Decadienal-*d*$_4$ ql	290.0	46.1	-95	-10	-82	-3
5	D-Fructose qn	314.0	236.0	-65	-10	-14	-27
5	D-Fructose ql	314.0	137.0	-65	-10	-32	-17
5-$^{13}C_6$	(IS) D-Fructose-$^{13}C_6$ qn	319.9	239.9	-110	-10	-14	-23
5-$^{13}C_6$	(IS) D-Fructose-$^{13}C_6$ ql	319.9	137.0	-110	-10	-28	-15
6	L-Glutamic acid qn[b]	263.0	136.9	-75	-10	-26	-15
6	L-Glutamic acid ql[b]	263.0	124.9	-75	-10	-22	-15
6-$^{13}C_5$	(IS) L-Glutamic acid-$^{13}C_5$ qn	268.1	136.9	-75	-10	-28	-15
6-$^{13}C_5$	(IS) L-Glutamic acid-$^{13}C_5$ ql	268.1	129.9	-75	-10	-22	-15
7	Hexanal qn	234.1	136.8	-80	-10	-26	-17
7	Hexanal ql	234.1	45.9	-80	-10	-92	-7
7-*d*$_{12}$	(IS) Hexanal-*d*$_{12}$ qn	246.1	137.8	-85	-10	-28	-13
7-*d*$_{12}$	(IS) Hexanal-*d*$_{12}$ ql	246.1	46.0	-85	-10	-82	-21
8	L-Lactic acid qn	223.9	136.9	-180	-10	-26	-9
8	L-Lactic acid ql	223.9	45.9	-185	-10	-74	-21
15	(IS) 3-Hydroxypropionic acid qn	224.0	194.0	-150	-10	-16	-17
15	(IS) 3-Hydroxypropionic acid ql	224.0	45.9	-125	-10	-60	-21
9	Methional qn	238.0	189.9	-55	-10	-16	-27
9	Methional ql	238.0	47.0	-55	-10	-54	-21
9-*d*$_3$	(IS) Methional-*d*$_3$ qn	240.9	189.9	-55	-10	-16	-21
9-*d*$_3$	(IS) Methional-*d*$_3$ ql	240.9	49.8	-55	-10	-48	-7
10	3-Methylbutanal qn	220.0	176.9	-90	-10	-22	-21
10	3-Methylbutanal ql	220.0	137.0	-90	-10	-22	-15
10-*d*$_2$	(IS) 3-Methylbutanal-*d*$_2$ qn	221.9	178.9	-90	-10	-22	-21
10-*d*$_2$	(IS) 3-Methylbutanal-*d*$_2$ ql	221.9	137.0	-90	-10	-22	-9
11	3-Methylbutyric acid qn	236.0	136.9	-80	-10	-30	-15
11	3-Methylbutyric acid ql	236.0	151.8	-110	-10	-22	-21
11-*d*$_2$	(IS) 3-Methylbutyric acid-*d*$_2$ qn	238.1	136.8	-85	-10	-28	-15
11-*d*$_2$	(IS) 3-Methylbutyric acid-*d*$_2$ ql	238.1	152.9	-85	-10	-24	-17
12	(*E*)-2-Nonenal qn	274.0	137.0	-140	-10	-28	-17
12	(*E*)-2-Nonenal ql	274.0	45.9	-140	-10	-84	-9
13	Phenylacetaldehyde qn	254.0	137.0	-70	-10	-26	-11
13	Phenylacetaldehyde ql	254.0	207.0	-70	-10	-18	-15
13-*d*$_5$	(IS) Phenylacetaldehyde-*d*$_5$ qn	259.1	137.0	-55	-10	-28	-11
13-*d*$_5$	(IS) Phenylacetaldehyde-*d*$_5$ ql	259.1	212.0	-55	-10	-18	-15
14	Vanillin qn	286.0	147.9	-5	-10	-28	-9
14	Vanillin ql	286.0	271.0	-5	-10	-22	-27
14-*d*$_3$	(IS) Vanillin-*d*$_3$ qn	289.1	150.9	-40	-10	-24	-17
14-*d*$_3$	(IS) Vanillin-*d*$_3$ ql	289.1	270.9	-40	-10	-18	-31

[a] qn = quantifier, ql = qualifier, [b] the sum of glutamic acid and pyroglutamic acid was determined due to the reaction occurring during the derivatization with 3-NPH (cf. Figure S3a).
